# Evidence to Support the Anti-Cancer Effect of Olive Leaf Extract and Future Directions

**DOI:** 10.3390/nu8080513

**Published:** 2016-08-19

**Authors:** Anna Boss, Karen S. Bishop, Gareth Marlow, Matthew P. G. Barnett, Lynnette R. Ferguson

**Affiliations:** 1 Discipline of Nutrition, FM & HS, University of Auckland Medical School, Private Bag 92019, Auckland 1142, New Zealand; MarlowG@cardiff.ac.uk (G.M.); l.ferguson@auckland.ac.nz (L.R.F.); 2 Auckland Cancer Society Research Centre, FM & HS, University of Auckland Medical School, Private Bag 92019, Auckland 1142, New Zealand; k.bishop@auckland.ac.nz; 3 Food Nutrition & Health Team, Food & Bio-based Products Group, AgResearch Limited, Grasslands Research Centre, Tennent Drive, Palmerston North 4442, New Zealand; matthew.barnett@agresearch.co.nz

**Keywords:** olive leaf, oleuropein, oxidative stress, inflammation, Mediterranean diet, Cyclooxygenase-2

## Abstract

The traditional Mediterranean diet (MD) is associated with long life and lower prevalence of cardiovascular disease and cancers. The main components of this diet include high intake of fruit, vegetables, red wine, extra virgin olive oil (EVOO) and fish, low intake of dairy and red meat. Olive oil has gained support as a key effector of health benefits and there is evidence that this relates to the polyphenol content. Olive leaf extract (OLE) contains a higher quantity and variety of polyphenols than those found in EVOO. There are also important structural differences between polyphenols from olive leaf and those from olive fruit that may improve the capacity of OLE to enhance health outcomes. Olive polyphenols have been claimed to play an important protective role in cancer and other inflammation-related diseases. Both inflammatory and cancer cell models have shown that olive leaf polyphenols are anti-inflammatory and protect against DNA damage initiated by free radicals. The various bioactive properties of olive leaf polyphenols are a plausible explanation for the inhibition of progression and development of cancers. The pathways and signaling cascades manipulated include the NF-κB inflammatory response and the oxidative stress response, but the effects of these bioactive components may also result from their action as a phytoestrogen. Due to the similar structure of the olive polyphenols to oestrogens, these have been hypothesized to interact with oestrogen receptors, thereby reducing the prevalence and progression of hormone related cancers. Evidence for the protective effect of olive polyphenols for cancer in humans remains anecdotal and clinical trials are required to substantiate these claims idea. This review aims to amalgamate the current literature regarding bioavailability and mechanisms involved in the potential anti-cancer action of olive leaf polyphenols.

## 1. Introduction

Cancer is a group of diseases involving proliferation of mutated cells [1]. In 2012, over 14 million new cases of cancer were reported [2], triggering a push to further develop treatments and preventative strategies. Cancer is predominantly an age-related disease, therefore with better conditions of life and increased longevity it is likely to continue increasing in prevalence. However, there are clearly factors other than age that contribute to its development. The traditional Mediterranean diet (MD) has gained robust scientific support for providing protection against some cancers [3,4]. The MD has shown an ability to influence the inflammatory response, which plays a pivotal role in aging and in reducing its age-associated non-communicable diseases such as cancer. However, the mechanisms of action behind the effects of the MD on inflammation are not entirely clear [5,6,7]. It has been suggested that the NF-κB inflammatory response, eicosanoid pathways and oxidative stress via free radical formation, have been suggested to play a role in MD related health benefits [5,8,9]. The diet, as a whole, has shown a protective role in cancer, however, the distribution of people still consuming it is gradually receding due to the spread of the western-type urban society, globalization and consumption [10]. Because of this, it is important to understand whether any beneficial effects ascribed to the MD are due to a particular component of the diet, rather than the whole diet. As one example, polyphenol bioactive components have shown particular promise and have therefore been a research focus. 

Extra virgin olive oil (EVOO) is typically used as a traditional component of the MD and has also been correlated with improved cardiovascular disease and cancer outcomes [11,12]. EVOO is manufactured by pressing olives to create a paste, which is churned to amalgamate oil droplets which are then extracted. There is a considerable variation in EVOO characteristics that can be attributed to the olive variety, the geographical location the olives were derived from [13] and the method of oil extraction [14]. Intake of both MD and EVOO has been shown to correlate with a reduced overall risk of cancer and is more specifically associated with reduced risk of cancers of the digestive system, prostate and breast [12].

**Figure 1:**
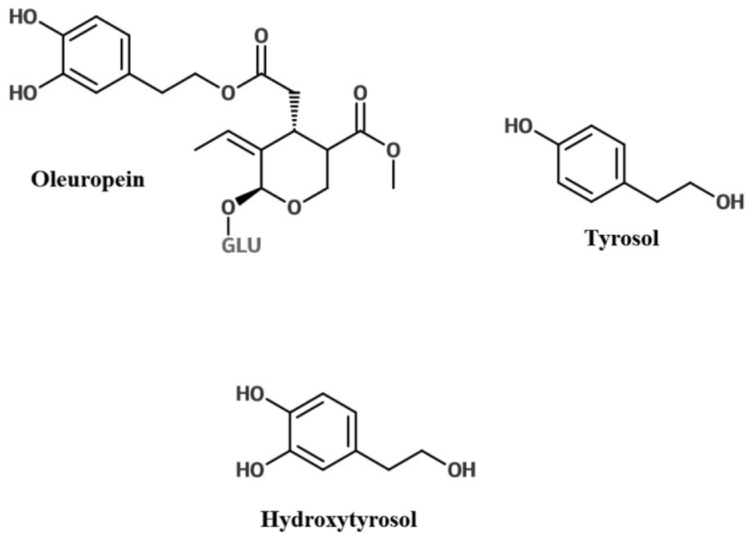
The olive polyphenol hydroxytyrosol and its derivatives, oleuropein and tyrosol (adapted from [19]).

EVOO is primarily a monounsaturated fatty acid (MUFA) in the form of oleic acid, with minor components including various phenolics [15]. It has been recognised that the polyphenol content plays an important role in health benefits. The European Food Safety Authority (EFSA) have approved the use of the general claim “olive oil polyphenols contribute to the protection of blood lipids from oxidative stress” when oil contains no less than 5 mg of hydroxytyrosol (HT) and its derivatives (such as tyrosol and oleuropein) per 20 mL OO [16] (Figure 1). There are several studies that have shown that EVOO with higher phenolic content provides stronger anti-inflammatory and antioxidant effects than OO with a lower phenolic content [17,18]. This suggests the phenolic component, rather than the fat in the oil, is the effector. 

**Figure 2:**
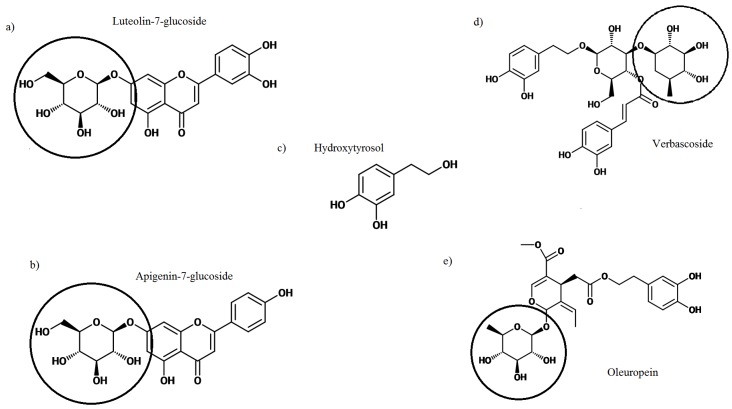
Most abundant phenolics present in OLE. Structures (**a**) and (**b**) are flavonoids. Structures (**d**) and (**e**) are esters of (**c**) which is a simple phenolic. The glucoside moieties are circled. This figure is adapted from [19].

Olive tree leaves (*Olea europaea*) are widely used in traditional medicine in the Mediterranean region [20]. In the Bible, the olive plant is referenced numerous times for its medicinal use [21]. The bioactive properties of the leaf have created a foundation for use as an antioxidant, anti-hypertensive, anti-atherogenic, anti-inflammatory, hypoglycemic, and hypocholesterolemic treatment [20]. Olive tree leaves contain similar polyphenols to those found in EVOO or the fruit itself, albeit at a much higher concentration [20,22]. Consequently, olive leaf extract (OLE) may hold an even greater potential than EVOO for improving health outcomes. During EVOO processing leaves can unintentionally be left in the mixture if the separation methods are inadequate, alternately leaves can also be added to EVOO mixtures to provide health benefits and improve flavor [23]. The addition of leaves increase the phenolic and chlorophyll content of the oil but also the organoleptic traits as measured in volunteer taste tests [24]. Components of OLE that are not detected in the oil from the fruit include several flavonoids, namely luteolin and apigenin, which have demonstrated anti-cancer properties [25,26,27,28,29]. In addition, the structure of phenolics differs between the olive fruit and leaf, with OLE containing a higher proportion with a glycoside moiety (Figure 2 and Table 1) [19]. The presence of a glucose molecule could play an important role in respect to both bioavailability and bioactive potential of the polyphenols, thereby impacting the health benefits for humans.

Although there is a large body of research that has investigated the phenolic components of olive products and the benefits they provide to human health [39,40,41,42], there are currently no approved claims in regard to OLE. OLE not only contains a higher quantity and variety of polyphenols than those found in EVOO, but many of the polyphenols also contain a glucose moiety. This structural difference in the polyphenols may have important consequences by altering their capacity to improve health outcomes [43,44]. In previous work, OLE polyphenols have demonstrated the ability to inhibit proliferation of several cancer cell lines including pancreatic [45], leukaemia [46] and breast [28,47]. Cellular models for breast and prostate cancers have been inhibited by the olive polyphenols oleuropein and HT [48,49,50,51]. Importantly, oleuropein and HT have consistently been reported to discriminate between cancer and normal cells; inhibiting proliferation and inducing apoptosis only in cancer cells. The intake of polyphenols in observational studies is difficult to quantify and therefore assign effect and intervention studies in regards to cancer have not been carried out, therefore the relationship between polyphenols and cancer outcomes in humans has not been substantiated.

Research into the anti-cancer properties of olive polyphenols is abundant with a focus on the health effects of EVOO. Evidence suggests that the bioactive components of OLE, although similar to EVOO, may be more potent and therefore show more potential for improving health outcomes. This review aims to amalgamate the current literature regarding bioavailability and anti-cancer mechanisms involved in OLE polyphenol action. The literature identified for this review was found using the search engines PubMed-NCBI, Scopus and ScienceDirect with a combination of block searching and pearl-growing. Key words used for the search were olive leaf extract, polyphenols, cancer, oleuropein, hydroxytyrosol, Mediterranean diet, inflammation, and bioavailability. The key components from the research articles pivotal to this review have been summarized in Supplementary Table S1.

## 2. Olive Leaf Polyphenols

The Mediterranean region, where olive trees are predominantly grown, is characterized by extended periods of sunlight and high rates of pathogen and insect attack. To combat these stressors, olive trees synthesize high volumes of polyphenols which are largely stored in their thick leaves [52]. The concentration and variety of polyphenols present in the leaves will be influenced by many factors such as geographical location, cultivar of tree, and the age of the tree [49]. Polyphenols comprise multiple phenolic groups, each consisting of an aromatic ring with a varying number of hydroxyl groups [19]. The polyphenols predominantly occur in a conjugated form, with one or several sugars attached to the hydroxyl group [53]. The number and structure of phenol rings in a polyphenol are used for classification and will determine its bioactive properties. The main phenolic compounds are the secoiridoids (namely oleuropein) and flavonoids (Figure 2), these have shown the ability to influence human and animal inflammatory and metabolic biomarkers [41,54,55,56].

Secoiridoids are a group of compounds found exclusively in plants of the *Olearaceae* family, and make up the majority of olive polyphenols (~85% of olive leaf polyphenols) [57]. In OLE the secoiridoid, oleuropein is the most abundant polyphenol (Figure 2), while its derivatives oleuropein aglycone, oleoside, and ligstroside aglycone are also present at varying concentrations [19]. The research surrounding oleuropein is abundant. It has been associated with numerous health benefits including the ability to: lower blood pressure in rats [58], decrease plasma glucose concentrations in rats [55], inhibit the growth of microbes grown on agar plates [59], inhibit cultured parasitic protozoans [60] and has also shown the ability to induce apoptosis in cancer cell models: colorectal [61], breast ([61,62,63] and prostate [48]. Human trials looking into the effect of OLE on cancer do not yet exist.

**Figure 3:**
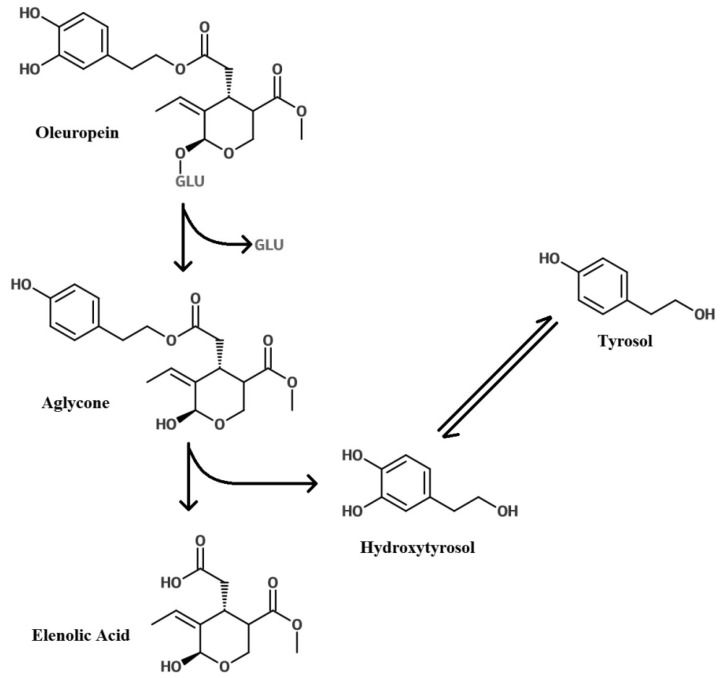
Glycosylation of oleuropein to its aglycone this gives rise to elenolic acid and hydroxytyrosol. Tyrosol in turn is hydrolysed from hydroxytyrosol (modified from Granados-Principal et al., 2010 [64]).

Hydrolysis of oleuropein gives rise to oleuropein aglycone, elenolic acid, HT and a glucose molecule (Figure 3) [64]. HT is a phenolic alcohol and the second most abundant phenolic acid in olive leaf. Tyrosol is another phenolic acid derived from oleuropein, but is found in low concentrations in the leaf (Table 1). Other related compounds include verbascoside, which also has demonstrated anti-inflammatory, anti-oxidant and antineoplastic properties similar to the other olive leaf bioactives [65], as well as caffeic acid (220.5 ± 23.3 mg/kg) [35] and p-coumaric acid.

OLE consists of a number of flavonoids (~2% of olive leaf polyphenols) including luteolin, apigenin (Table 1), rutin (495.9 ± 12.2 mg/kg) [35], catechin (19.3–32.6 mg/g dried extract) [66] and diosmetin (8.70 mg/g dried extract) [22]. Luteolin is able to suppress inflammatory expression in macrophages and adipocytes [67]. Apigenin is present at relatively low concentrations within olive leaf, but it has also been linked to anti-inflammatory, anti-cancer and anti-oxidising properties [68]. 

Other components of OLE that occur in smaller concentrations include oleanolic acid [69], vanillin and vanillic acid, [59], as well as tocopherols and β carotene [70]. In human studies, α tocopherols have been correlated to lower prostate cancer mortality, but β carotene at high concentrations, has been correlated to increased mortality of lung cancer patients [71]. 

Thousands of phytochemicals with differing attributes have been identified and isolated, but a point which is often overlooked is that it can be a combination of compounds that induce health benefits [72,73,74]. Within plants, polyphenols are present in mixtures and not as independent compounds; the polyphenols have evolved together, generally for the purpose of deterring insect feeding and the levels of the different bioactives with these mixtures need to be considered when looking at bioactive properties for human health. While the evolutionary purpose for the polyphenol mixtures it not for human benefit, the nature of the mixtures may nevertheless be important for human health. Several studies have demonstrated that the phenolic compounds from OLE may display a synergistic effect when in the same proportions as occurring naturally in the olive leaf. The secoiridoids, flavonoids and other phenols in OLE provide a stronger anti-microbial and antioxidant effect when working together, as opposed to the phenolics independently [59,75,76]. Through the use of different antioxidant assays it was determined that OLE flavonoids, simple phenols and secoiridoids utilize different mechanisms to exert an anti-oxidant effect [75], which at least in part explains their additive effect.

## 3. Bioavailability of Olive Leaf Polyphenols

In nutrition, bioavailability refers to the amount of compound/nutrient extracted from a food or supplement that is capable of being absorbed and made available for physiological use by the body [77]. There are many factors that will influence the bioavailability of a compound including the vector, time taken for absorption, structure of compound/bioactive target or the individual person [78]. The matrix that the olive leaf is consumed and maintained may also have an impact on the bioavailability of the active components. The leaves can be consumed in tea, as a powder or in an extract form As an example, De Bock and co-authors demonstrated that the polyphenol derivatives measured in plasma differed when the OLE was administered as a safflower oil compared to a glycerol matrix [79]. 

The ability to produce health benefits in different organs throughout the body requires that the bioactive olive leaf polyphenols, or their metabolites, are able to infiltrate these areas. After an acute load of olive phenolic (3 g phenolic extract from olive cake/kg of body weight) extract in mice, samples demonstrated that phenolic derivatives and conjugates (oleuropein, tyrosol, HT and luteolin) were absorbed, metabolised and present in the plasma (oleuropein derivative: max 4 h: 24 nmol/L and HT: max 2 h: 5.2 nmol/L), the heart (luteolin derivative at 1 h: 0.47 nmol/g), kidney (luteolin derivative 1 h: 0.04 nmol/g, HT max 4 h: 3.8 nmol/g), testicles (olueropein derivative Cmax 2 h: 0.07 nmol/g and HT max 2 h: 2.7 nmol/g) and had even passed the blood brain barrier (olueropein derivative at 2 h: 2.8 nmol/g) [80]. 

The research looking into bioavailability of polyphenols from OLE in commercial glycerol formulations consistently show that oleuropein is bioavailable in humans but there is differing evidence regarding the metabolites found in plasma [79,81]. De Bock reported the primary metabolite recovered to be glucoronidated and sulphated HT [79]. In contrast, Kendall’s group reported that no HT was detected in urine samples, but glucuronic acid conjugates, derived from oleuropein aglycone were detected [81]. In rats fed oleuropein, liquid chromatography-mass spectrometry (LC-MS) detected oleuropein, oleuropein aglycone, elenolic acid and HT both within faeces and urine at 24 h [82]. This demonstrates the stability of these compounds and therefore the potential ability to reach other parts of the body intact and in an active form. 

Corona et al. (2006) reported HT and tyrosol traversed the perfused small intestine membrane of rats but oleuropein did not, and would therefore likely reach the large intestine intact [83]. Incubating with anaerobic human microbiota with olueropein resulted in rapid and extensive microbiota degradation of oleuropein to HT and other metabolites [83]. Specifically the gastrointestinal bacterium *Lactobacillus planatarum* has the ability to metabolize oleuropein to HT [84]. The microbiota acting to break down oleuropein to HT would have an important impact on bioavailability if oleuropein cannot traverse membranes, but HT and other metabolites can, as reported by Corona et al. 2006. Another study has since found that oleuropein orally administered to rats resulted in the production of oleuropein metabolites from the gastrointestinal tract as well as metabolites in the blood [82]. The most recent research looking into the metabolism of oleuropein verses oleuropein aglycone in rodents (5 mg phenol/kg/day) found that oleuropein resulted in the greatest bioavailabilty (measured by the highest content of HT excreted in urine) and a greater diversity of microbial metabolites due to its superior ability to reach the colon intact [44]. 

### Glycosylation of Polyphenols

The glucose moiety that is present on many of the olive leaf polyphenols could have an important impact on their bioactive properties. The glucose molecule significantly increases the molar mass of the polyphenol; oleuropein is 540.51 g/mol, where the oleuropein aglycone is 394 g/mol. The glucose molecule may improve stability and bioavailability, and facilitate cell entry but it also may impede bioactive properties.

Through collection and processing methods of olives and leaves, different glycosylation enzymes are activated [85]. The transformation of oleuropein is dependent on the type of glycosylation enzyme acting (β-glucosidase, hemicellulase, tannase, neutral protease, cellulase, glucoamylase, papain, alkaline protease, amylase, β-glucanase) and this will result in varied concentrations and ratios of HT, oleuropein aglycone, elonolic acid and total phenolics [86,87]. The combination of polyphenols may improve the OLE biostability, insuring polyphenols are still present in the olive leaf extract when consumed by humans but also improving the polyphenols ability to reach different areas of the body intact. For example oxidoreductase enzymes reduce the abundance of oleuropein in OLE, but the presence of HT is able to inhibit their action [86].

Olive leaf polyphenols containing a glucose moiety have been suggested to play an important role in relation to cancer cell treatment. A study looking at oleuropein found removal of the glucose moiety reduced its ability to inhibit proliferation of cancer cells [43]. This indicated that the hydrophilic glucose may be enabling oleuropein to enter cells via GLUT transporters to create the anti-cancer affect. GLUT mRNA expression is often increased in cancer cells and is correlated to cancer progression [88]. The glucose moiety in oleuropein may facilitate its diffusion into these cells in precedence to normal cells and therefore result in a greater inhibitory effect on cancer versus normal cells. Another study has indicated that the olive flavonoid apigenin is able to reduce the expression of GLUT1 in prostate cancer cell lines thereby inhibiting proliferation of the cancer [29]. 

Another study looking at the effect of oleuropein (dissolved in water) verses oleuropein aglycone (dissolved in ethanol 100%) (6 to 100 µM) in MCF-7 found the aglycone to be more effective at reducing cell viability [89]. This would suggest that the glycoside is essential for anti-cancer effects.

Protective effects of the MD and EVOO against cancers, as discussed in the introduction, are primarily associated with cancers of the digestive system. This could be due to the bioavailability of the polyphenols, with the polyphenol constituents creating the anti-cancer effects not being able to reach other parts of the body to have an impact. Consequently if the glucose moiety, a prominent characteristic of olive leaf polyphenols improves bioavailability it may also improve protective effects for different cancers. 

## 4. OLE and Evidence of the Ability of Olive Leaf Polyphenols to Scavenge Nitric Oxide and Quench Reactive Oxygen Species

Reactive oxygen species (ROS) and nitrogen species (NOS) are essential for cell function. They are involved in energy supply, detoxification, chemical signaling and immune response. However, when overproduced they can create stress by damaging DNA, lipids and proteins and they are widely accepted to play an important role in pathologies and aging [20,90]. Chronic disease is associated with oxidative stress, therefore an increased antioxidant intake or intake of compounds that enhance the body’s own antioxidant system is expected to reduce the risk of these diseases. It was this hypothesis that has led to an increased interest in antioxidants and their bioactive properties. Phenolics are one group for which there is robust evidence supporting the health promoting effects of antioxidants. There is a general consensus that olive leaf phenolics have a strong ability to scavenge nitric oxide (NO) and quench ROS [91,92]. 

**Figure 4:**
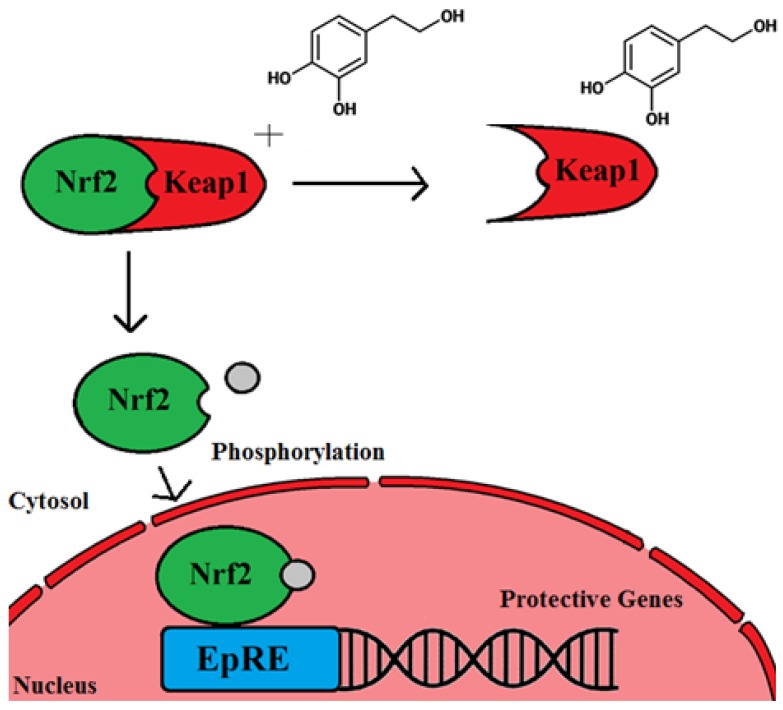
Polyphenol interaction with Nrf2 and activation of *EpRE* genes. The polyphenol (HT) reacts with Keap1 permitting Nrf2 to escape. Nrf2 requires phosphorylation before it is able to enter the nucleus. This schematic is modified from [93].

Antioxidant properties have been an important focus of research into polyphenols and are a widely accepted mechanism for their health benefits. However, it has been suggested that several constraints impede polyphenol in vivo scavenging of radicals, and that they would be inefficient at mounting an antioxidant defense [93]. Concerns that have been highlighted include bioavailability (the anti-oxidizing agent must reach these radicals in an active form to quench them) and kinetic constraints for antioxidant scavenging (radicals may actually react with other biological molecules such as DNA and lipids in the cell at the same rate as the antioxidants) [93]. This could mean that a very high concentration of polyphenols would need to be ingested to perceive any effect in humans. Instead it is suggested that antioxidant compounds, such as polyphenols, are able to activate transcription factors such as nuclear factor (erythroid-derived 2)-like 2 (Nrf2) that bind to the Electrophile Response Element (EpRE) and thereby transcribe genes for protective enzymes that provide the health benefits (Forman et al., 2014 and Figure 4). Several in vitro studies using humans cells and animal in vivo studies investigating olive polyphenols have supported Nrf2 activation and its consequential expression of protective genes [72,94]. Conversely, a recent human intervention study has shown no evidence of altered phase II enzyme expression (the downstream product of Nrf2 activation) in peripheral blood mononuclear cells following consumption of HT (5 mg and 25 mg per day in olive mill waste water) [95]. The olive mill waste water was tested to confirm oleuropein was not present.

The Xenohormesis hypothesis suggests the stress-induced secondary metabolite production in plants is recognized by humans upon consumption, and these signals initiate stress response pathways [72,73]. Similarities in the human and plant extracellular signal-regulated kinase (ERK) pathways (these are able to activate many transcription factors and play an important role in cell regulation functions) show that polyphenols are able to activate pathways, such as AMP-activated protein kinase (AMPK) and hold the potential to modulate redox and mitochondrial signaling [96,97]. During eukaryotic evolution, glucose was the preferred carbon source. Rapid cell growth was the best way to utilize glucose, and AMPK activation provided the off switch mechanism in this process [72]. Therefore, AMPK activation (or similar pathways) could result in decreased ATP and increases in mitochondrial free radicals, implicating protection from chronic disease and aging [72]. Evidence for this theory was provided by microarray analysis of gene expression after EVOO treatment of breast cancer cells. These results demonstrated up-regulation of AMPK, and the top Canonical pathway regulated was the Nrf2 Mediated Oxidative stress pathway [72].

HT in vitro studies using human cell lines has been shown to up-regulate the expression of endogenous antioxidant genes (Heme Oxygenase 1 (HO-1), NAD(P)H-quinone oxidoreductase (NQO1), Glutathione (GSH)) via Nrf2 overexpression. The c-Jun N-terminal kinase (JNK) pathway plays an important role in inflammatory signaling. The JNK pathway was up-regulated following treatment with HT and inhibiting this pathway established its requirement for GSH and *p62* regulation. However, *HO-1* or *NQ-1* were unaffected [94]. p62 inactivates *Keap1*, increasing Nrf2 in the nucleus and consequently increasing the expression of oxidation defense enzyme genes [98]. Oleuropein in a human in vitro model has also been shown to activate Nrf2 and HO-1 expression [99]. However, in vivo human trials with HT have failed to find an up-regulation of phase 2 enzymes which are the by-product of EpRE and Nrf2 stimulation [95].

## 5. Olive Leaf Properties That Protect against Development and Progression of Cancer

Genetic changes are involved in the prevalence of cancers, however it is environmental and lifestyle factors such as obesity [100], unbalanced diet, tobacco, lack of exercise and alcohol consumption that account for the majority of the attributing cause [101]. Olive leaf contains strong anti-oxidants, it would be logical to conclude that these would help in mitigating the effect of genetic lesions that give rise to cancer. However, olive leaf has also attracted attention as a potential cancer treatment [28,46,102,103]. In previous work, olive leaf polyphenols have demonstrated the ability to inhibit the proliferation of several cancer cell lines including pancreatic [45], leukaemia [46], breast [28,47,49], prostate [48] and colorectal [61]. Importantly, oleuropein and HT have consistently been reported to discriminate between cancer and normal cells; inhibiting proliferation and inducing apoptosis only in cancer cells [48,49]. The challenge with relating the anti-cancer effects in cell models to in vivo arises when considering bioavailability of the polyphenols. This could explain why OO protective effects in humans show a strong association with cancers of the digestive system [12]. In other cancers OO phenolics has been suggested to act as phytoestrogens and anti-inflammatory agents, thus producing a protective effect.

A higher risk of breast cancer is linked to over-exposure to oestrogen [104,105] and growth of breast cancer can be stimulated by estradiol, which binds to the oestrogen receptor (ER). This receptor is an important biomarker and target for breast cancer prevention and treatment [106]. Work with breast cancer cell lines and OLE polyphenols have indicated potential mechanisms of action that include action as a phytoestrogen. Oleuropein and HT both possess an aromatic ring that is similar to that in estradiol, therefore these compounds are hypothesized to compete with oestrogens for receptor binding sites [50,107]. In the MCF-7 breast cancer cell line, HT and oleuropein (at doses between 10 and 75 μM) dose-dependently prevented cell proliferation through inhibition of the oestrogen activated ERK1/2 signaling pathway but did not show a direct effect on the mediation of ER gene expression [50]. It was later shown that oestrogen responses were also mediated by the GPER/GPER30 receptors, of which HT and oleuropein are agonists [108]. Despite both oestrogen and the polyphenols showing the same mechanism of receptor binding, they have opposite effects. Oestrogen leads to cell proliferation, while polyphenols lead to apoptosis or cell death. Both activate the ERK1/2 pathways but it has been proposed that the length of activation could influence the effect, with prolonged activation leading to apoptosis, and short-term to cell proliferation [108]. Sustained ERK activation has previously been demonstrated to result in inhibition of MCF-7 cell growth [109]. In vivo studies looking at olive leaf polyphenols also appear to support an anti-cancer effect. Oleuropein (125 mg/kg of diet) slowed tumor growth and inhibited cancer metastasis after MCF-7 cell xenograft establishment in mice [110]. OLE dissolved in water (150 and 225 mg/kg/day) reduced tumour volume and weight in mice after breast cancer xenograft [111].

The aromatase (CYP19) enzyme is the catalyst for the rate determining reaction in oestrogen synthesis. Inhibiting CYP19 effectively prevents oestrogen synthesis and because high levels of oestrogen are linked to breast cancer, this holds potential as a treatment [112]. A recent clinical study has shown that amylase inhibitors taken daily for 5 years were successfully able to reduce the incidence of breast cancer in high-risk postmenopausal women [113]. In MCF-7 cells, luteolin suppressed CYP19 transcription potentially via activator protein-1 (AP1) and C/EBP binding to the aromatase promoter [26]. 

The olive flavones apigenin and luteolin have been shown to act as aryl hydrocarbon receptor (AhR) antagonists in mouse cell lines [114]. Upon ligand binding, AhR is translocated to the nucleus where it activates response elements in the DNA sequence and consequent production of xenobiotic enzymes [115]. Other work has found that AhR in cancer cell lines acts as a tumour suppressor through diminished DNA replication and G0/G1 arrest [116]. Another study has reported that apigenin suppresses the growth of MCF-7 cells, inhibiting the NF-κB signaling pathway, the phosphorylation of IkBα, and nuclear translocation of p65 within the nucleus [27]. Apigenin was not found to inhibit cell survival signaling through mediators such as AKT, ERK, JNK, or p38, but it decreased STAT3 transcriptional activity in the cells, indicating that this compound induces growth-suppressive activity. The transcription factor STAT3 is more specifically involved in inflammatory signaling within cancer tumours and interacts with cytokines [117], thus by inhibiting STAT3, luteolin could also be having an anti-inflammatory effect. In another study oleuropein was cytotoxic to MDA-MB-231 and MCF-7 cells, avoiding damage to normal cells, with apoptosis taking place via induction of the mitochondrial pathway [49]. MCF-7 cell proliferation was inhibited by oleuropein at the S-phase of the cell cycle by an up-regulation of the p21 gene, and inhibition of NF-κB and its target D1 gene expression.

In PC3 and DU145 prostate cancer cell lines, HT has demonstrated the ability to interfere with cell proliferation [51]. HT also activated mitogen-activated protein kinase (MAPK), ERK, p38 MAPK and JNK. However, when inhibited by specific antagonists, HT was still able to inhibit cell growth. The authors concluded that HT was able to induce apoptosis in cancer cells via the generation of superoxide dismutase (SOD) and extracellular ROS.

Work using the prostate cancer cell lines, LNCaP and DU145, found that oleuropein was pro-oxidative, causing loss of viability, but in non-malignant cells (a benign hyperplastic prostatic epithelial cell line) oleuropein acted as an anti-oxidant [48]. The downstream products of EpRE activation were all increased with oleuropein; pAkt, y-glutamylcysteine (y-GCS), heme oxygenase-1 (HO-1) and ROS. Interference with pAkt was proposed as the mechanism enabling cell apoptosis in these prostate cancer cell lines [48]. 

### 5.1. Anti-Inflammatory Properties of Olive Leaf Polyphenols and Their Effects on Cancer

Inflammation is the natural defense mechanism against foreign threats, and its mechanisms are essential for survival. However, chronic inflammation, even at low levels, has been correlated to many health complications and age-associated diseases, including but not limited to cancer and cardiovascular disease [118]. The NF-κB signaling pathways play a pivotal role in inflammatory response and are an attractive target for preventing inflammation. NF-κB resides inactive within the cytoplasm due to the presence of IκB kinase, an inhibitor enzyme, therefore it can be activated very quickly to initiate cytokine and prostanoid production. There is strong evidence that olive polyphenols are able to interact with these pathways [119,120,121]. 

**Figure 5:**
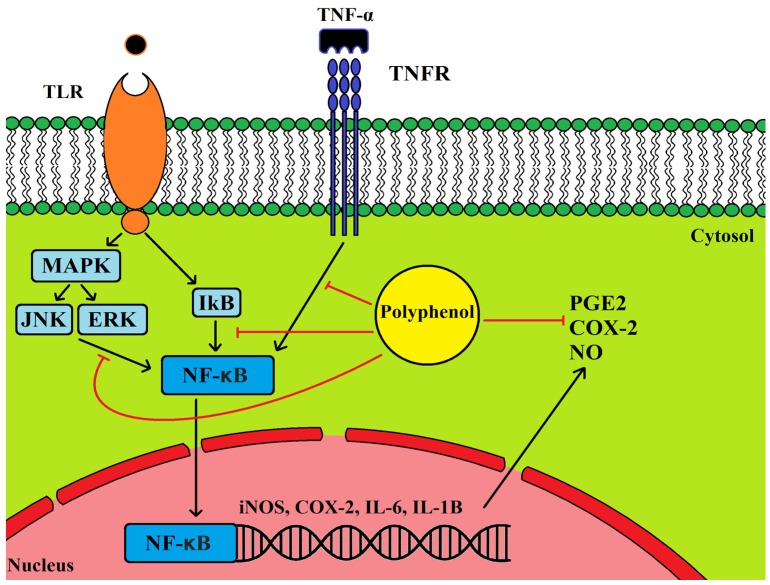
Olive leaf polyphenols may interact with gene and protein expression directly or via an interaction with receptors on the cell membrane. Toll-like receptor (TLR) and tumour necrosis factor receptor (TNFR) activation results in inflammatory gene expression (COX2, IL-6, IL-6 and IL-1β) and prostanoid production. This illustration shows the potential points at which OLE polyphenols could interact if able to enter the cell membrane.

The cyclooxygenase 2 (COX-2) enzyme plays an important role in inflammation as the catalyst for the synthesis for prostanoids and hence an inflammatory response [122]. Cellular studies with OLE polyphenols have found a protective effect in relation to inflammation; a down-regulation of NO and COX-2 [120,123,124,125]. Inhibition of the Toll-like receptor (TLR) signaling induced by LPS was demonstrated not only by down-regulation of iNOS and COX2, but also by a decrease in ERK1/2, JNK and nuclear factor of kappa light polypeptide gene enhancer in B-cells inhibitor alpha (IκBα) phosphorylation in vitro after oleuropein treatment [120] (Figure 5). In down-regulating this pathway the pro-inflammatory enzymes interleukin 6 (IL-6) and interleukin 1β (IL-1β) and the gene AP-1 were also down-regulated. In human monocytes HT inhibited LPS induced COX-2 and prostanoid production, however, it increased TNF-α. In contrast in human cell models tyrosol down-regulated TNF-α and induced NF-κB, JNK and ERK phosphorylation and COX-2 expression [126] (Figure 5). Lastly the olive flavonoid luteolin regulated IL-1β induced COX-2 expression via ERK, JNK and NF-κB [127].

### 5.2. Cancer, Inflammation and COX2 Expression

An overexpression of COX-2 has been linked to invasiveness of many cancers including human breast cancer [128,129], prostate [130] and colorectal [131]. Drugs that inhibit COX-2 enzymes are able to reduce the risk of breast cancer [132], and have pro-apoptotic effects in the MCF-7 cell line [133] and prostate cancer cell lines [134]. Luteolin, when administered with the COX-2 inhibitor celecoxib, created a synergistic effect in MCF-7 and three other breast cancer cell lines. Interestingly, the ERK1/2 levels were inhibited in the oestrogen receptor positive cell lines, but were increased in the negative cell lines [135]. Down-regulation of the phosphatidylinositide 3-kinase (P13K)/Akt pathway inhibits phosphorylated Akt levels, which in turn stimulates apoptosis. Phosphorylated Akt levels were decreased in all cell lines [135].

A review on breast cancer found all stages of cancer progression corresponded to COX-2 expression [129]. COX-2 is a down-stream product of NF-κB which was down-regulated in MCF-7 treated with oleuropein [49]. In mouse models, COX-2 driven prostaglandin E2 (PGE2) expression in mammary tissue led to an increase of *CYP19* and aromatase-catalysed oestrogen biosynthesis [136]. Samples taken from patients with breast cancer showed a correlation between transcription of *CYP19* and both gene and protein expressions of COX-2 and PGE2 [137]. In a previous study the authors hypothesized that HT and oleuropein were able to inhibit proliferation via competing for oestrogen binding sites [50]. These studies suggest that OLE polyphenols may be acting in MCF-7 to block oestrogen receptor binding and to inhibit COX-2 expression, which appears to down-regulate *CYP19* expression [136]. 

Another gene that COX-2 can regulate is p53. Work in human mammary tissue has demonstrated that COX-2 represses p53 transcription thereby inhibiting cell apoptosis [138] and it has since been demonstrated that p53 down-regulates aromatase expression in breast adipose stromal cells [139]. Work looking at the effects of oleuropein in MCF-7 has shown that it is able to induce apoptosis via up-regulating p53, and consequently the transcription of Bax/Bcl-2 apoptotic genes [62]. Other studies have also measured a change in p53 and Bax expression with oleuropein inhibition of cervical cancer cells [140] and p53 pathway up-regulation with oleuropein inhibition of colorectal cancer cells [61].

In vivo, luteolin (10 mg/kg/day) reduced both volume and weight of tumors in a prostate xenograft mouse model and in vitro, using the prostate cancer cells PC-3, it down-regulated VEGF phosphorylation of VEGF2 receptor and its downstream inflammatory markers IL-8 and IL-6 [25]. If VEGF is correlated to PGE2, as in the breast cancer models mentioned above, then it could be a downstream effect of COX-2 inhibition. 

PGE2 expression pushes the immune response from a T-helper 1 (Th1) (including cells such as Natural killer (NK) cells) to a Th2 (such as mast cells) and Th17 mediated response, which is less effective at fighting off infections or protecting from cancer [141]. This potentiates acute, local inflammation driven by phagocytes, which is less aggressive than the Th1/Th17 response [141]. By down-regulating COX-2, the balance will shift back to Th1, which may improve immune-competence. For example COX-2 knock out in breast cancer cells inhibited tumour growth by enhancing T-cell survival and immune surveillance in tumours [142].

The tumour microenvironment has an important impact on tumour progression and metastasis, therefore its manipulation has been suggested as a target for cancer therapy [143]. It has been demonstrated in breast cancer MCF-7 cells that tumour associated macrophages are able to enhance COX-2 levels in the tumour. Conversely inhibiting COX-2 in macrophages was able to inhibit levels in the tumour [144]. In several human intervention studies with olive polyphenols, COX-2 expression in immune cells was down-regulated [145,146]. In cancer patients this could potentially lead to a down-regulation of COX-2 in tumours and thereby inhibit tumour progression. In other intervention studies the inflammatory markers NF-κB, p65, IKKβ, and IKKα [147] and NF-κB, IL-6 and IL-1β [148] have been down-regulated with olive polyphenols. These studies measured changes after single 40 mL doses of EVOO (containing the olive polyphenols), quantities achievable in an individual's standard diet.

### 5.3. Quinone Hypothesis for Anti-Cancer Properties of Olive Leaf

As quinones, olive leaf polyphenols could bind to the cysteine residues of NF-κB in cancer cells and manipulate gene expression. This would explain the observed gene expression in in vitro models [46,120,126]. A recent study has indicated olive leaf polyphenols in a quinone form could interact with Topoisomerase IIα [149]. The olive leaf polyphenols oleuropein, verbascoside, and HT were categorized by Vann et al. as Topoisomerase IIα poisons. Topoisomerase IIα is an enzyme essential for cell survival, catalysing the breaking and re-joining of the DNA helix to remove tangles and playing an important role in cell replication. Acting as Topoisomerase IIα poisons the polyphenols increased DNA cleavage, this effect was 10–100 times stronger in the presence of an oxidant [149]. This is consistent with the idea that the polyphenols have been transformed into quinone electrophiles, which are then able to bind to cysteine residues. This study also demonstrated that the olive leaf polyphenol tyrosol was unable to act as a poison consistent with its inability to form a quinone and bind to the cysteine residue within Topoisomerase IIα. 

Although potentially dangerous in normal cells, Topisomerase IIα is an important target for cancer treatment. Due to the requirement of an oxidant environment, this might explain why no toxicity has been shown in normal cells in comparison to tumour cell models; the quinones were not formed. 

## 6. Conclusions

There is strong evidence from cell models which demonstrates that olive polyphenols, and specifically the combination found in olive leaf, are able to modulate and interact with molecular pathways and in doing so may inhibit the progression and development of cancer. However, it is important to acknowledge that cell models are very different from the complex human body and applying these findings to cancer outcomes in humans is difficult. 

Meta-analysis correlating the consumption of a MD and OO in humans to protection from digestive system, prostate and breast cancers [4,12], suggest that the effects may be constrained by bioavailability but also directs to a phytoestrogenic mechanism of action. Not only are the reduced risk of oestrogen related cancers in females correlated to protective effects of phytoestrogens, but a recent meta-analysis has correlated a lower risk of prostate cancer with phytoestrogen consumption [150].

The evidence suggests that olive polyphenols may act differently when in different combinations and at different concentrations. The presence of a glucose molecule, one factor that differentiates olive leaf polyphenols from OO polyphenols, is likely to affect the bioavailability and therefore bioactive properties. Changes to microbiota and microbiota-mediated degradation of polyphenols, demonstrate the glucose molecule has an effect. 

Both cell models and human intervention studies demonstrate olive polyphenols are creating an anti-inflammatory change involving NF-κB inhibition. The down-stream products of NF-κB: including COX-2, IL-6, IL-8, IL-1β are expressed at lower levels creating a tumour micro-environment that no longer facilitates progression or development of cancers. This may account for the lower prevalence of cancer in people consuming a MD. 

To answer the question “does OLE protect against cancer?” is difficult. Evidence is available in cell and animal models to support the conclusion that OLE does have beneficial effects and there is anecdotal evidence that olive polyphenols have a protective effect against cancer in humans. People consuming the MD have a lower prevalence of cancer, the MD consists of a high content of polyphenols, and olive leaf is an excellent source of many of these polyphenols. However, in order to prove that OLE improves cancer outcomes in humans, clinical trials would be required. 

## Figures and Tables

**Table 1:** Comparison of phenolic compounds found in olive leaf extract and olive oil, with values reported in mg/kg [30]. Luteolin, apigenin, verbascoside and oleuropein all have a glucoside moiety. Values are an estimated range generated from a comprehensive review of the published literature.

	Hydroxytyrosol	Oleuropein	Luteolin-7-Glucoside	Apigenin-7-Glucoside	Verbascoside	Oleuropein Aglycone	Reference
Olive oil mg/Kg	131.77 ± 32	ND	ND	ND	ND	17.24 ± 1.15	[30]
	3.0 ± 0.2	ND	ND	ND	0.08 ± 0.02	NM	[31]
	12.5	ND	NM	NM	NM	NM	[32]
	4.3–9.9	ND	4.0–7.6	1.5–2.6	ND	67.7–136.4	[33]
	0.15–1.53	ND	ND	ND	ND	0.35–6.43	[34]
Olive leaf mg/Kg	NM	26,471.4 ± 1760.2	4208.9 ± 97.8	2333.1 ± 74.7	966.1 ± 18.1	NM	[35]
	ND	19,050 ± 880	155 ± 10	207 ± 10	1428 ± 46	NM	[31]
	NM	19,860 ± 54	NM	NM	200 ± 40	NM	[36]
	NM	22,610 ± 632	970 ± 43	1072 ± 38	488 ± 21	NM	[37]
	NM	5173–12,921	219–444	192–488	213–501	NM	[38]

Abbreviations: not detected: ND; not measured: NM.

## Supplementary Materials

The following are available online at http://www.mdpi.com/2072-6643/8/8/513/s1, Table S1: Olive leaf polyphenol treatment in different cancer models; in vivo and in vitro.

## Abbreviations

The following abbreviations are used in this manuscript:

AhR	Aryl hydrocarbon receptor
AP1	Activator protein-1
EVOO	Extra Virgin Olive oil
HT	Hydroxytyrosol
JNK	c-Jun *N*-terminal kinase
MD	Mediterranean diet
MAPK	Mitogen-activated protein kinase
Nrf2	Nuclear factor (erythroid-derived 2)-like 2
NO	Nitric oxide
OLE	Olive leaf extract
OO	Olive oil
ROS	Reactive oxygen species
TLR	Toll-like receptor
