# Supplementary material for: Evidence to Support the Anti-Cancer Effect of Olive Leaf Extract and Future Directions
Source: Nutrients. 2016 Aug 19;8(8):513. doi: 10.3390/nu8080513 (PMC4997426; doi:10.3390/nu8080513)
Supplement: Supplementary file 1 [file nutrients-08-00513-s001.docx]

Supplementary Materials: Evidence to Support the Anti-Cancer Effect of Olive Leaf Extract and Future Directions

Anna Boss, Karen S. Bishop, Gareth Marlow, Matthew P. G. Barnett and Lynnette R. Ferguson

**Table S1.** Olive leaf polyphenol treatment in different cancer models; in vivo and in vitro.

| **Cancer** | **OLE Polyphenols** | **In Vivo/In Vitro** | **Effect** | **Reference** |
| --- | --- | --- | --- | --- |
| Pancreatic | OLE of Corregiola and Frantoio variety *Olea europaea* L. leaves in water, 50% ethanol or 50% methanol | MiaPaCa-2 | Anti-proliferative with 100–200 μg/mL, similar effect with all solvent matrices | [1] |
| Breast | Luteolin | MCF-7 cells | Suppressed CYP19 mRNA expression, inhibition potentially involving JNK and ERK pathways | [2] |
|  | OLE | JIMT-1 | Strong anti-proliferative effects (IC_50_: 7 μg/mL), partially mediated by ERK1/2 pathway inhibition. | [3] |
|  | OLE | SKBR3 | 200 μg/mL cytotoxic to cells | [4] |
|  | Oleuropein | MDA-MB-231 and MCF-7 cells | 200–300 μM induced apoptosis via the mitochondrial pathway | [5] |
|  | HT Oleuropein | MCF-7 | 10–75 MM, dose dependent prevention of proliferation via inhibition of ERK1/2 pathway | [6] |
|  | HT Oleuropein | MCF-7 | Apoptosis via a p53-dependent pathway mediated by Bax and Bcl2 genes. | [7] |
|  | Olueropein | In vivo MCF-7 xenograft in mice | 125 mg/kg of diet inhibited tumor growth and cancer metastasis. | [8] |
|  | OLE powder in distilled water | In vivo spontaneous mouse mammary tumor transplanted in mice | 150 and 225 mg/kg/day of OLE reduced tumor weight in mice and increased oxidative markers | [9] |
| Prostate | HT | PC-3 cells | 80 µmol/L caused apoptosis via mitochondrial dysfunction and activated MAPK kinases | [10] |
|  | Luteolin | In vivo prostate xenograft mouse model PC-3 cells | 10 mg/kg/day reduced weight and volume of tumours.  Down-regulated VEGF phosphorylation and IL-8 & IL-6 in prostate cells. | [11] |
|  | Oleuropein | LNCaP, DU145 | Pro-oxidative causing loss of cell viability in prostate cells with down-stream products of EpRE measured; GSH, ROS, HO-1. | [12] |
| Colorectal | Oleuropein HT | HT-29 cells | Apoptosis, up-regulation of p53 protein but no changes in IkB-α and MAPK cascade protein expressions. | [13] |
| Leukemia | OLE | Human chronic Myelongenous Leukemia K562 | 100 μg/mL and 150 μg/mL induced apoptosis & monocyte/macrophage differentiation | [14] |

References

1. Goldsmith, C.D.; Vuong, Q.V; Sadeqzadeh, E.; Stathopoulos, C.E.; Roach, P.D.; Scarlett, C.J. Phytochemical Properties and Anti-Proliferative Activity of *Olea europaea L*. Leaf Extracts against Pancreatic Cancer Cells. *Molecules* **2015**, *20*, 12992–3004.
2. Li, F.; Ye, L.; Lin, S.; Leung, L.K. Dietary flavones and flavonones display differential effects on aromatase (CYP19) transcription in the breast cancer cells MCF-7. *Mol. Cell. Endocrinol.* **2011**, *344*, 51–58.
3. Barrajón-Catalán, E.; Taamalli, A.; Quirantes-Piné, R.; Roldan-Segura, C.; Arráez-Román, D.; Segura-Carretero, A.; Micol, V.; Zarrouk, M. Differential metabolomic analysis of the potential antiproliferative mechanism of olive leaf extract on the JIMT-1 breast cancer cell line. *J. Pharm. Biomed. Anal.* **2015**, *105*, 156–162.
4. Quirantes-Piné, R.; Zurek, G.; Barrajón-Catalán, E.; Bäßmann, C.; Micol, V.; Segura-Carretero, A.; Fernández-Gutiérrez, A. A metabolite-profiling approach to assess the uptake and metabolism of phenolic compounds from olive leaves in SKBR3 cells by HPLC-ESI-QTOF-MS. *J. Pharm. Biomed. Anal.* **2013**, *72*, 121–126.
5. Elamin, M.H.; Daghestani, M.H.; Omer, S.A.; Elobeid, M.A.; Virk, P.; Al-Olayan, E.M.; Hassan, Z.K.; Mohammed, O.B.; Aboussekhra, A. Olive oil oleuropein has anti-breast cancer properties with higher efficiency on ER-negative cells. *Food Chem. Toxicol.* **2013**, *53*, 310–316.
6. Sirianni, R.; Chimento, A.; De Luca, A.; Casaburi, I.; Rizza, P.; Onofrio, A.; Iacopetta, D.; Puoci, F.; Andò, S.; Maggiolini, M.; et al. Oleuropein and hydroxytyrosol inhibit MCF-7 breast cancer cell proliferation interfering with ERK1/2 activation. *Mol. Nutr. Food Res.* **2010**, *54*, 833–840.
7. Han, J.; Talorete, T.P.N.; Yamada, P.; Isoda, H. Anti-proliferative and apoptotic effects of oleuropein and hydroxytyrosol on human breast cancer MCF-7 cells. *Cytotechnology* **2009**, *59*, 45–53.
8. Milanizadeh, S.; Bigdeli, M.R.; Rasoulian, B.; Amani, D. The Effects of Olive Leaf Extract on Antioxidant Enzymes Activity and Tumor Growth in Breast Cancer. *Thrita* **2014**, *3*, doi:10.5812/thrita.12914.
9. Osborne, C.; Tripathy, D. Aromatase inhibitors: Rationale and use in breast cancer. *Annu. Rev. Med.* **2005**, *56*, 103–116.
10. Luo, C.; Li, Y.; Wang, H.; Cui, Y.; Feng, Z.; Li, H.; Li, Y.; Wang, Y.; Wurtz, K.; Weber, P.; et al. Hydroxytyrosol promotes superoxide production and defects in autophagy leading to anti-proliferation and apoptosis on human prostate cancer cells. *Curr. Cancer Drug Targets* **2013**, *13*, 625–639.
11. Pratheeshkumar, P.; Son, Y.-O.; Budhraja, A.; Wang, X.; Ding, S.; Wang, L.; Hitron, A.; Lee, J.-C.; Kim, D.; Divya, S.P.; et al. Luteolin inhibits human prostate tumor growth by suppressing vascular endothelial growth factor receptor 2-mediated angiogenesis. *PLoS ONE* **2012**, *7*, e52279.
12. Acquaviva, R.; Di Giacomo, C.; Sorrenti, V.; Galvano, F.; Santangelo, R.; Cardile, V.; Gangia, S.; D’Orazio, N.; Abraham, N.G.; Vanella, L. Antiproliferative effect of oleuropein in prostate cell lines. *Int. J. Oncol.* **2012**, *41*, 31–38. Available online: http://www.spandidos-publications.com/ijo/41/1/31/abstract (accessed 21 May 2015).
13. Hassan, Z.K.; Elamin, M.H.; Omer, S.A.; Daghestani, M.H.; Al-Olayan, E.S.; Elobeid, M.A.; Virk, P. Oleuropein Induces Apoptosis Via the p53 Pathway in Breast Cancer Cells. *Asian Pac. J. Cancer Prev.* **2013**, *14*, 6739–6742.
14. Samet, I.; Han, J.; Jlaiel, L.; Sayadi, S.; Isoda, H. Olive (*Olea europaea*) Leaf Extract Induces Apoptosis and Monocyte/Macrophage Differentiation in Human Chronic Myelogenous Leukemia K562 Cells: Insight into the Underlying Mechanism. *Oxid. Med. Cell. Longev.* **2014**, *2014*, 927619.
